# Post-discharge symptoms following fast-track colonic cancer surgery: a phenomenological hermeneutic study

**DOI:** 10.1186/2193-1801-3-276

**Published:** 2014-06-02

**Authors:** Marianne Krogsgaard, Pia Dreyer, Ingrid Egerod, Mary Jarden

**Affiliations:** Digestive Disease Center, Bispebjerg Hospital, Copenhagen University Hospital, Bispebjerg Bakke 23, DK-2400 Copenhagen, NV, Denmark; Faculty of Health, Aarhus University, Noerrebrogade 44, building 21,1, DK-8000 Aarhus, Denmark; Department of Anesthesia and intensive Care Medicine, Aarhus University Hospital, Noerrebrogade 44, building 21,1, DK-8000 Aarhus, Denmark; Faculty of Health and Medical Sciences, University of Copenhagen, Blegdamsvej 9, DK-2100 Copenhagen, Denmark; Copenhagen University Hospital Rigshospitalet, Trauma Centre, Blegdamsvej 9, DK-2100 Copenhagen, Denmark; The University Hospitals Centre for Health Research, Rigshospitalet, Ryesgade 27, DK-2200 Copenhagen, N, Denmark

**Keywords:** Colonic cancer, Early discharge, Fast-track programme, Nursing, Postoperative recovery, Qualitative research, Symptom management

## Abstract

**Objective:**

To obtain knowledge of patients’ experiences of postoperative symptoms during the initial two weeks following fast-track colonic cancer surgery.

**Method:**

Semi-structured in-depth interviews with seven colonic cancer patients two weeks post hospital discharge. Analysis was performed using a phenomenological hermeneutical approach.

**Results:**

During the first two weeks after discharge the patients experienced unfamiliar symptoms that affected their everyday lives. Despite distressing symptoms, they applied a “wait-and-see” strategy, and only reacted when symptoms became intolerable. The patients failed to report their unfamiliar symptoms during hospital nurse follow-up telephone call. While waiting for the final histology patients suffered loss of sleep and chaotic thinking, and experienced ambiguity of hoping for the best and expecting the worst.

**Conclusion:**

Although fast-track surgery programmes lead to shorter hospitalisation and improved physical performance, post-colonic surgery patients experience various symptoms after discharge. Healthcare professionals need to address symptoms that might have immediate and long-term consequences on patients’ everyday life. Follow-up studies are encouraged to explore the patient perspective to identify the needs of individual patients after hospital discharge.

## Background

Implementation of multimodal fast-track programmes has increased internationally since the concept was introduced in the early 1990s (Kehlet 1997; Ansari et al. 2013). The main components of fast-track surgery are patient information, surgical stress reduction, pain management, adequate nutrition and early mobilization (Kehlet and Dahl 2003; Fearon et al. 2005). The fast-track colonic surgery programme comprises: a preoperative consultation including patient information and a cancer diagnosis, short-term hospitalization with surgery and post-surgical care, post-discharge follow-up phone call by hospital nurse, and outpatient consultation two weeks after discharge including the final histology that indicates the need for further treatment.

After the introduction of standardised evidence-based colonic cancer surgery, the hospital length of stay has been reduced from 8–12 days in conventional elective resections, to 2–3 days in fast-track programmes, without an increased readmission rate (Wind et al. 2006; Kehlet 2008). The resulting early discharge leaves patients to handle their postoperative symptoms and a cancer diagnosis without professional assistance (Kehlet and Mogensen 1999; Basse et al. 2001; Fearon et al. 2005). Postoperative symptoms such as pain and fatigue are frequently reported, but difficulties involving anxiety, lack of sleep, nausea, and altered bowel function are also reported within the first two weeks following colonic surgery (Henriksen et al. 2002; Zutshi et al. 2005; King et al. 2006; Mohn et al. 2009; Wennstrom et al. 2010; Kahokehr et al. 2011).

A recent meta-synthesis described the negative impact of symptoms in cancer patients, increasing their need for optimal communication and preparatory information regarding treatment-related symptoms (Bennion and Molassiotis 2013). One of the key features of fast-track surgery is the provision of extensive preoperative information preparing patients for active participation in the multimodal regimen of treatment and care to meet selected goals for early discharge and resumption of normal activities after discharge (Kehlet 2008). More knowledge is needed into the patient perspective to provide optimal care. The aim of the study was to obtain knowledge of patients’ experiences of postoperative symptoms during the initial two weeks following fast-track colonic cancer surgery.

## Methods

The study is a qualitative phenomenological hermeneutic study based on semi-structured interviews with cancer patients participating in a fast-track surgery programme. To study the patient’s experiences of everyday life we used a semi-structured interview guide with open-ended questions (Kvale and Brinkmann 2009). Interpretation of the interview text is based on Lindseth and Nordberg’s (Lindseth and Norberg 2004) phenomenological hermeneutic approach which builds upon the French philosopher Paul Ricoeurs’ theory of interpretation (Ricoeur 1973b). Ricoeur stated that “what is to be interpreted in a text is what it says and what it speaks about” (Ricoeur 1973a). The researcher seeks to explain and understand the meaning of life experiences as told by participants. According to Ricoeur interpreting a text means seeing something new in what is already taken for granted, and disclosing a sort of being-in-the-world (Ricoeur 1973a).

### Participants

The sample included seven colonic cancer patients participating in a fast-track surgery programme at a Danish University Hospital. The study was conducted at the Digestive Disease Center where fast-track programmes have been practised for the past 10–12 years. Inclusion criteria were patients with cancer, who had undergone fast-track colonic resection without colostomy and discharged from the hospital by the 4^th^ postoperative day. This criterion was selected pragmatically. Although the median length of stay is three days, 48% of patients are discharged on the 4th postoperative day or later (Stottmeier et al. 2011). Exclusion criteria were inability to read, write or speak the Danish language and residence on the Island of Bornholm due to time-consuming transportation. Seven patients were consecutively recruited the day before or after surgery from February to April 2012. Of twelve consecutive eligible patients, five were excluded: four from Bornholm and one male that refused participation.

Participant characteristics are presented in Table 1. All but one patient had laparoscopic surgery, none of the participants received nursing care at home, and none had children living at home. Physical performance improved from discharge to two weeks postoperatively, with one patient able to carry out all pre-operative activities without restriction (Eastern Cooperative Oncology Group, ECOG = 0) within two weeks postoperatively. At discharge, patients were provided with direct unit telephone numbers and were encouraged to contact the nurses if they had questions or concerns. Usual care included a follow-up phone call by the hospital nurse one to two days after discharge.

**Table 1:** **Participant characteristics**

Gender	Age	Co-habitation	Discharge day*	ECOG at discharge	ECOG at interview	Day of interview**	Adjuvant treatment	Employment status
Female	55	yes	2	2	1	14	yes	Working
Female	59	yes	2	2	1	12	no	Working
Female	64	no	3	3	1	14	no	Retired
Female	74	yes	2	2	1	13	no	Retired
Female	79	no	3	2	1	15	yes	Retired
Male	82	yes	2	2	0	14	no	Retired
Male	82	yes	2	2	1	14	no	Retired

ECOG = The Eastern Cooperative Oncology Group Performance. ECOG score from 0–5 (0 = fully active, 5 = death).

Adjuvant treatment = Chemotherapy starting 2–3 weeks postoperatively. Discharge day* = days after surgery. Day of interview** = days after surgery.

### Data collection

Eligible patients were introduced to the study by the study nurse (MK), who was not already known to the patients. Written informed consent was obtained during hospitalization and patients were later contacted for interview appointment by telephone (MK). Interviews were conducted at the location selected by the participants, being in their own home (n = 4), in a private room at the hospital (n = 2) or at the workplace (n = 1). The seven patients that consented to participate were already enrolled in our on-going quality of life study at the hospital using the self-reported questionnaire European Organisation for Research and Treatment of Cancer Quality of life Core 30 (EORTC QLQ-C30). Scores regarding symptom distress one and two weeks postoperatively were included in the last segment of the interview to prompt patients’ memory in the present study. Patients’ physical performance was scored using ECOG performance status at discharge and at the time of interview (Oken et al. 1982). The semi-structured interviews were conducted two weeks postoperatively by the first author (MK), had an average length of 64 minutes (range 38–83 min) and were transcribed verbatim.

A semi-structured interview guide with open-ended questions explored how patients resumed everyday life, including their views on information provided by professionals regarding symptoms, and their own symptom experience and coping strategies. We posed open-ended questions such as: “What was important to you in order for you to resume everyday life?” and “How did you experience symptoms? Each interview started with a broad question letting the informant speak freely, e.g. “Tell me about your first day at home after discharge”. This allowed for unexpected experiences to emerge (Kvale and Brinkmann 2009). To enhance trustworthiness the interviewer was an open and attentive listener and avoided interpretations during the interview. To ensure reliability, transcription was carried out verbatim by the interviewer, keeping as close to what was said as possible without subjective interpretation.

### Ethical considerations

The patients were introduced to the study by the first author (MK) on the day of admission and were given 24 hours to consider participation. The patients received written and verbal information on the purpose of the study, including the right to withdraw from the study and assurance of confidentiality according to the basic principles for research stated in the Helsinki Declaration and Northern Nurses federation (Northern Nurses’ Federation (NNF) ( 2003)). The study was registered at the Danish Data Protection Agency (j.no.2012-41-0160).

### Data analysis

Influenced by Ricoeurs interpretation theory Lindseth and Nordberg argue for an in-depth analysis of text using a dialectic method on three levels (Lindseth and Norberg 2004). Several other nurse researchers have applied this method of analysis and subscribe to explicit principles for the analysis (Kouwenhoven et al. 2012; Wenstrom et al. 2012; Dellenmark-Blom and Wigert 2013). Based on these views, our analysis was performed on three levels: naïve reading, structural analysis, and critical analysis and discussion. Naïve reading is the first impression of the interview text as a whole, achieving an immediate understanding of the content. Structural analysis is carried out on three levels: “what is said” (quotes), “what the text speaks about” (meaning), and finally, the text is structured into main themes and sub-themes (interpretation). In critical analysis, the interpretation continues with a discussion of findings in a dialectic pattern between explanation and comprehension achieved by relating to the naïve reading, the validated themes, and external literature relevant for the topics. This “final act of comprehension” is used to argue in favour of one or several suitable interpretations of the text (Ricoeur 1973b).

In our study, the findings emerged through analysis and interpretation among a group of qualitative investigators discussing the relationship between the parts and the whole text, increasing the credibility and trustworthiness of our findings (Lindseth and Norberg 2004; Polit and Beck 2012). Selected quotes from participants are presented to allow the reader to judge interpretations and add credibility to the analysis. Data were managed using the qualitative computer software package NVivo version 9.0 (QSR international Pty Ltd., Victoria, Australia), which created an audit trail, providing credibility and reliability to the findings.

## Findings

### Naïve reading

The naïve reading was our initial spontaneous impression of how the patients experienced postoperative symptoms expressed as a meaning of the whole. After discharge, patients experienced their symptoms as unfamiliar, causing worry or doubt and affecting everyday life. Despite difficulties in interpreting symptoms, the patients did not contact the hospital. They relied on their everyday experiences and their relatives to relieve symptoms. The patients actively regained control over usual daily activities despite experiencing persistent or sudden symptoms that limited activity. Psychological recovery was a considerable part of convalescence; the cancer diagnosis stunned the patients and caused them grief. While waiting for the final histology, they experienced lack of sleep and chaotic thinking. Waiting was described as a time of uncertainty, affecting everyday life and recovery. Patients experienced ambiguity as they hoped for the best and prepared for the worst.

### Structural analysis

The structural analysis entailed interpretation of the text in dialectic movements between quotes and the interpreted meaning (Table 2). The analysis resulted in two main themes and their sub-themes (Table 3).

**Table 2:** **Example of structural analysis**

Direct quote	Meaning	Interpretation
***“What is said”***	***“What the text speaks about”***	Main theme: symptoms permeate daily life
It really is odd that I feel so tired. I wonder what my blood count is. There may be something wrong, but as my son said: Mom you are 74, it might just be something ordinary.	At home patients experienced unfamiliar symptoms causing worry and doubt. Patients interpreted their symptoms alone or with relatives seeking explanations.	Symptoms cause concern
I kept thinking oh, no… is it a thrombosis or a stroke because I had sensory disturbances. I wondered if it was stress.		
Suddenly I felt a pressure in my stomach and I lost my appetite. It seemed strange to me, as it ought to be the other way around; with a lack of appetite first…I thought that it would soon disappear, so I could start eating again. I am so skinny.	Sudden or persistent symptoms limited activities in everyday life. Sudden fatigue required frequent rest.	Symptoms limit activity
During the first week I was suddenly overcome by fatigue and had to rest for 20 minutes before I could continue with what I was doing.		
The first days at home I did nothing at all because of pain. I only put on underwear and a sheet to avoid the pain.		
We went for a walk, just a short one; I mean we don’t have to walk for hours as usual. Then I went to bed again and slept for an hour. Every day I went to bed after breakfast and again after lunch, I was so tired.		

Structural analysis was conducted on three sub-levels: “what is said” (quotes), “What the text speaks about” (meaning), and final structure into main themes and sub-themes (interpretation).

**Table 3:** **Main themes and sub-themes**

Main themes	Symptoms permeate daily life	Experience of grief
Sub-themes	• Daily activities are driven by own motivation	• Stunned by cancer diagnosis
	• Symptoms limit activity and cause concern	• Chaotic thinking
	• Symptoms are managed by own experiences	

### Main theme: symptoms permeate everyday life

#### Subtheme: daily activities driven by own motivation

Once at home, patients resumed their usual physical activities like going for a walk, bicycling, shopping, or returning to work. Some were compelled to return to daily life, and driven by their impetus to recover, patients focused on their resources. One woman was determined to resume her seven kilometre walk to work:

*“I have to be able to walk to work, so I told myself walk 1000 steps today, 2000 tomorrow… I set goals for myself…. I have an idea that my willpower is important. If you are determined to recover, you’ll recover more easily”. (Female, 55).*

Other patients were less determined and proceeded step-by-step to regain pre-illness level of activity. Relatives had no major influence on the patients’ accomplishment of physical activities, but instead they helped with daily tasks as cooking and shopping. Patients gradually resumed more demanding activities like doing laundry or cleaning. They were, however, still physically limited due to weakness and fatigue.

#### Subtheme: symptoms limit activity and cause concern

Patients experienced various symptoms such as fatigue, sleeplessness, loss of appetite, urinary symptoms, lack of concentration, diarrhoea, scrotal oedema, pain and sensory disturbances. Unfamiliar symptoms caused worry and doubt about cause, duration and consequences. As one patient said:

*“Suddenly I felt pressure in my stomach, and I lost my appetite. It seems strange to me, as it ought to be the other way around, losing my appetite first*… *I expected it to disappear, so I could start eating again. I am so skinny”. (Female, 79).*

Difficulty in recognizing common postoperative symptoms caused anxiety and some patients started suspecting cancer and speculated whether the tumour had been removed:

*“… the diarrhoea I had to endure the first week took its normal course. By the second week I found it very troublesome, very. The irregularity of my bowel movement was the reason I saw a doctor in the first place. Therefore I worried whether they got it all [tumour] out?”. (Male, 82).*

The patients employed various strategies to manage their symptoms. Some patients waited for the bowel function to normalize, applying a “wait-and-see” strategy. They considered contacting the hospital, but refrained because they believed symptoms were temporary and they wanted to avoid causing concern in the family. A woman with untreated cystitis for two weeks said:

*“…Well, I didn’t think that it was anything worth calling about. Now I remember the nurse telling me, don’t call if you have a swollen finger. Then I thought, is a bladder infection worth calling about? I just waited until Monday [outpatient appointment]”. (Female, 79)*.

During a follow-up call from the hospital a few days after discharge the patients did not mention their symptoms. One patient in particular told the nurse that he was doing well, although he had uncomfortable symptoms. Symptoms limited activities in everyday life and the patients responded when they reached their physical limit, e.g. being overcome by fatigue, in persistent pain, or needing sleep. As a consequence daily walks were cut short and other activities such as cooking and cleaning were avoided.

#### Sub-theme: symptoms are managed by own experiences

Patients were concerned about their symptoms and sought information on the Internet, consulted their relatives, or relied on their own experience. A woman suffering loss of appetite said:

*“…my son recommended…a certain chocolate bar. He said, go buy some and cut them into small bites and eat them. Well, it might be a good idea” (female, 64).*

In some cases lack of knowledge of a symptom led to disagreement between the patient and family regarding when to call the hospital. The reason to avoid calling could be fear of the consequences or just not wanting to disturb the nurses. A woman that lived alone had a friend staying for the week and said:

*“One day I felt really bad, and my friend said contact them [the unit]. I said, forget it. She wanted me to call them. I told her no, come on, I’ll go to bed, there is no need to disturb them”. (Female, 64).*

### Main theme: experience of grief

#### Sub-theme: stunned by cancer diagnosis

Although the patients still did not have a final histology, they were stunned by the cancer diagnosis. The patients described their trajectory from perfect health, to awareness of body changes, and to obsessing about a tumour:

*“Normally I feel that I am very much in touch with my body and listen to the signals it sends me. That is why it came as such a shock that I had a tumour”. (Female, 59).*

Immediately after the medical examination some patients were unprepared and shocked by being diagnosed with a tumour, while others had a notion it might be cancer. The patients grieved the loss of their prior life before the possible cancer:

*“When I think of March 10*^*th*^*in the morning, afternoon and part of the evening, I was blissfully unaware. Then I’m reading the paper to see what’s on television and all of a sudden I have to go to the toilet again, and then it doesn’t matter anymore what’s on TV. I knew something was seriously wrong”. (Female, 79).*

#### Sub-theme: chaotic thinking

While waiting for the pathology report, patients wondered whether the surgery had cured them. Ambiguous and chaotic thoughts balanced between hoping for the best and preparation for the worst:

*“I’ve had extreme reflections and questions like: What if I die? How will I endure a difficult treatment as chemo [therapy] or surgery? What will happen?”. (Female, 59).*

Some chose not to think or talk about their concerns, while others discussed their situation with relatives. Patients were more open with other cancer survivors who understood their situation. While waiting for biopsy results patient reactions were described as: “my mind was spinning”, “lying awake at night” or “difficulty finding myself”. Anxiety forced patients to find ways to distract their attention:

*“I’ve been anxious about the result. I’ve never watched that much television as in the past week to divert my thoughts”. (Female, 64).*

## Discussion

This study aimed at obtaining knowledge of patients’ experiences of postoperative symptoms during the first two weeks following fast-track colonic cancer surgery. Our main findings were that symptoms persisted and affected the everyday lives of patients after returning home from the hospital, and that the patients experienced grief as they lost their former life without cancer.

What sets this study apart from studies of conventional treatment is the fact that patients are discharged earlier and are left to cope with their symptoms in the context of daily life at home. Our study showed that patients needed to resume their normal activities and that they were driven by their own motivation. Resuming a familiar lifestyle is in accordance with other descriptions of postoperative recovery, demonstrating the importance of regaining control over habitual physical and social functioning, when returning to preoperative levels of independence (Allvin et al. 2007). The patients in our study succeeded in resuming everyday activities despite an array of worrying and uncomfortable postoperative symptoms. Bandura argues that a strong sense of self-efficacy is created through mastery of experiences (Bandura 1994); thus we assume it is important that health professionals encourage patients to resume familiar activities in the postoperative recovery process.

Patients in our study experienced diverse symptoms. While pain, fatigue and changes in bowel function are well described, other symptoms like oedema, concentration difficulties, sleeplessness and sensory disturbances are sparsely reported and might be under-studied. The experience of unfamiliar symptoms is in accordance with other studies of fast-track and conventional colorectal surgery (Blazeby et al. 2009, Barthelsson et al. 2008; Wennstrom et al. 2010; Jonsson et al. 2011). We assume that the symptoms were less upsetting to the patients when they were experienced in hospital during conventional surgery programmes where experienced staff was available.

In other populations, such as cardiac surgery, patients have reported symptoms that were not recorded in the standard questionnaires (Savage 1999). This demonstrates the importance of qualitative studies that inform clinicians and questionnaire developers of known and unknown symptoms that impact everyday life.

In our study, most symptoms were experienced as indeterminate or uncharacteristic and led to a “wait-and-see” strategy. According to Leventhal et al. ( 1997), weak or ambiguous symptoms are difficult to interpret and lead to longer assessment and delay in reporting symptoms (Leventhal et al. 1997; Leventhal et al. 2001). We assume this might explain why patients failed to handle symptoms in accordance with preparatory information, such as when to contact the unit if symptoms of urinary tract infection or diarrhoea arise. Some studies show that patients contact the hospital before the next scheduled appointment; however the symptoms reported are not clearly described in these studies (Norlyk and Harder 2011; Taylor and Burch 2011). In spite of worry and doubt regarding symptoms the scheduled outpatient appointment in the fast-track programme reassured patients and served as a time frame for patients needing professional assistance regarding symptoms. Consequently, short-term impact of the experienced symptoms seemed of minor importance. However other studies have found that 5–6% of patients are readmitted on the 5^th^ postoperative day after fast-track colonic cancer surgery (Stottmeier et al. 2011; Delaney et al. 2012) due to e.g. vomiting, urinary tract infection, bleeding and ileus (Stottmeier et al. 2011). Thus a “wait-and-see” strategy might be unsuitable for patients who are scheduled for follow-up appointments after two weeks.

According to Fearon et al. ( 2005) early discharge in fast-track programmes implies more active supervision of the patient when at home to ensure the patient is well (Fearon et al. 2005). Telephone contact is one way to access the patient’s situation. In our study patients failed to report their symptoms to the nurse during telephone follow-up, but they did, however, discuss their symptoms with relatives. Other studies have described these findings (Leventhal et al. 1997; Norlyk and Harder 2011) and it might be inferred that telephone follow-up is inadequate (Bennion and Molassiotis 2013). Patients need information to recognize symptoms that require medical attention (Bennion and Molassiotis 2013) and Cox and Wilson ( 2003) argue that the knowledge and skills of nurses are essential for assistance in telephone follow-ups (Cox and Wilson 2003).

According to our findings, it is important that professionals encourage patients to report both expected and unfamiliar symptoms during follow-up. When symptoms are ambiguous and diffuse, the follow-up nurse needs to explore the patient experience in a systematic manner. Further, exploring which symptoms the patient has discussed with relatives might improve patient openness in symptom reporting. However evidence is still lacking as to the best time, structure and method to conduct follow-up telephone calls (Mistiaen and Poot 2006; Cusack and Taylor 2010). Patients in our study discussed persistent symptoms at the outpatient appointment and were treated for urinary tract infection and diarrhoea. However if recognized at telephone follow-up unnecessary symptom distress might have been avoided.

The patients in our study were satisfied with the preparatory information and regarded the discharge information they received about common symptoms as adequate and relevant. It was reassuring to know that the unit could be contacted at any time for expert advice and support. However, once at home even expected symptoms were difficult to interpret and cope with, such as diarrhoea*,* fatigue and loss of appetite. When unable to use the information, patients relied on their everyday experiences and relatives as described in other studies (Williams 2008; Pedersen et al. 2012; Norlyk and Martinsen 2013).

We assume that information given in our study was too general to accommodate the individual patients and their symptoms. Preparatory information might reduce distress (Bennion and Molassiotis 2013) but individualized information is important to improve patient response (Fredericks et al. 2009). With this in mind, patients’ prior experiences should be incorporated in the preparatory individualized information provided by nurses. Even though there is limited time available due to short-term hospitalizations in fast-track programmes, preparing the patient for post-discharge recovery by rehearsing matters of importance regarding symptom management could promote patient self-efficacy.

The patients in our study were vulnerable during the diagnostic phase and after discharge. At home, awaiting results from the biopsy, life was kept “on hold”, as also described by Giske et al. ( 2009). Psychological recovery was an important part of the postoperative recovery in our study. The vulnerability and distress appeared to affect the patient perception of and response to symptoms and must be taken into account. As postoperative recovery is an energy-consuming process involving physical, psychological, social and habitual functions (Allvin et al. 2007), the process does not only include physical symptoms as described in fast-track information.

### Study limitations

The transferability of this study is limited to the context of fast-track colonic cancer surgery and to contemporary conditions as regimens change over time. Variation in gender, age and marital status might have broadened the findings, but the timing of the interview and the in-depth patient perspective complement and nuance the existing knowledge about patients’ perceptions of postoperative symptoms after discharge after fast-track colonic surgery. As a consequence of our criteria to exclude patients discharged after four days we have limited the scope of the study. Knowledge about these patients might inform clinical practice and future studies in fast-track colonic surgery.

## Conclusion

Although fast-track programmes lead to shorter hospitalisation and improved physical performance in patients undergoing colonic surgery, the patients still experience a variety of ambiguous symptoms during the first two weeks following surgery. The symptoms described in our study are not all covered in the preparatory information for fast-track colonic surgery. Healthcare professionals need to address symptoms that might affect the patients’ everyday life after hospital discharge. Future studies are encouraged to explore the patient perspective to identify the needs of individual patients after hospital discharge.
